# Pre‐irradiation dose requirements for reference‐class ionization chambers: Quantifying measurement stability

**DOI:** 10.1002/acm2.70273

**Published:** 2025-09-29

**Authors:** Takumi Yamada, Kazuki Mayumi, Satoshi Tanabe, Takayuki Nishikata, Naoki Kinoshita, Tatsuya Sakai, Hironori Sakai, Tetsuya Oka, Hiroyuki Ishikawa, Tsutomu Kanazawa

**Affiliations:** ^1^ Section of Radiology Department of Medical Technology Niigata University Medical and Dental Hospital Niigata Japan; ^2^ Department of Radiation Oncology Niigata University Medical and Dental Hospital Niigata Japan; ^3^ Division of Radiology Nagaoka Red Cross Hospital Niigata Japan; ^4^ Department of Radiological Sciences Faculty of Medical Science Technology Morinomiya University of Medical Sciences Osaka Japan; ^5^ Department of Radiology and Radiation Oncology Niigata University Graduate School of Medical and Dental Sciences Niigata Japan

**Keywords:** chamber type dependence, ionization chamber stability, output reproducibility, pre‐irradiation, reference dosimetry

## Abstract

**Background:**

Pre‐irradiation of ionization chambers is widely recommended to stabilize output readings for reference dosimetry in radiation therapy. However, the specific pre‐irradiation requirements for different chamber models, particularly under strictly controlled conditions that isolate chamber performance, remain unclear.

**Purpose:**

This study aimed to quantitatively evaluate the pre‐irradiation dose required to stabilize the output of various reference‐class ionization chambers, while minimizing the influence of environmental and system‐level uncertainties.

**Methods:**

Twelve reference‐class ionization chambers (Exradin A12, A1SL; IBA CC13, FC65G; PTW 30013, 31021) were evaluated under strictly controlled conditions (room temperature: 25  ±  1°C, water temperature: 25.00  ±  0.01°C, relative humidity: approximately 50%). Output stability was assessed using a 10 MV photon beam from a TrueBeam linac. Chamber readings were measured using an RT521R2 electrometer and normalized to an adjacent external monitor chamber. Readings are defined as stable if the reading variation is <0.1% and does not exhibit any trending. The optimal number of pre‐irradiation exposures and corresponding doses were determined for each chamber.

**Results:**

For the large‐volume chambers (Exradin A12, IBA FC65G, PTW 30013), output variation was within ±0.05% from the first irradiation, suggesting that pre‐irradiation may be unnecessary. In contrast, the middle‐volume class ionization chambers (Exradin A1SL, IBA CC13, PTW 31021) exhibited variations of up to ±0.25%, and required up to 100 MU × 25 (approximately 21.25 Gy) of pre‐irradiation to achieve stability under the controlled experimental conditions of this study.

**Conclusions:**

This study demonstrated that the required pre‐irradiation dose varies depending on the type of ionization chamber, and that the output variation is significantly lower than the previously reported 1%. These results may help streamline clinical reference dosimetry by tailoring procedures to each chamber.

## 1 INTRODUCTION

In radiation therapy, high reproducibility and reliability of dose measurements are essential, requiring both the stability of measurement devices and the standardization of measurement conditions. In clinical practice, reference dosimetry is used for linear accelerator (linac) output calibration, typically involving ionization chambers, extension cables, and electrometers.

Pre‐irradiation has been widely recommended to ensure stable dose readings.^1^, ^2^, ^3^, ^4^, ^5^, ^6^, ^7^ For example, the IAEA TRS‐398 recommends a pre‐irradiation dose of approximately 10 Gy to achieve charge equilibrium among internal chamber materials for reference‐class ionization chambers.^1^ Similarly, the AAPM TG‐51 Addendum reports that applying more than 10 Gy of pre‐irradiation can reduce chamber history‐related uncertainty to approximately 0.1%, while omitting this step may result in relative errors of up to 1%, depending on the chamber type.^3^

These recommendations are often based on the behavior of the entire dosimetry system. Few studies have attempted to isolate and evaluate the contribution of individual components – such as the linac, ionization chamber, and electrometer – to output stability. McEwen et al. previously reported pre‐irradiation response curves for several ionization chambers (PTW models 30012, 31010, and 31014), highlighting differences in performance depending on chamber type.^4^ However, these data were reported over 15 years ago. Recent improvements in chamber materials and construction may have altered their stability characteristics. In addition, operational variables such as usage frequency and cumulative dose have not been systematically investigated.

Quantitatively determining the pre‐irradiation dose required for ionization chambers commonly used in clinical practice could enhance the efficiency and reliability of routine dosimetry procedures. In particular, evaluating the effects of other factors – such as device aging and cumulative usage – may help establish evidence‐based criteria for daily quality assurance and contribute to the development of standardized, reproducible calibration workflows.

TRS‐398 also notes that sufficient time must be allowed for the dosimetry system to reach thermal equilibrium. Depending on the electrometer type, power may need to be kept on for more than 2 h. Additionally, after changing polarity or the applied voltage, up to 30 min or a dose exceeding 10 Gy may be required for stabilization, depending on the specific ionization chamber and conditions.^1^

This study aimed to quantitatively evaluate the pre‐irradiation dose required for several reference‐class ionization chambers with varying clinical usage histories. All measurements were conducted under conditions designed to minimize external uncertainties, with particular attention given to maintaining stable temperature and humidity for the water phantom, ionization chambers, and electrometer. The evaluation also considered chamber type and the impact of applied voltage changes on output stability.

## 2 MATERIALS AND METHODS

### 2.1 Selection of ionization chambers

A total of twelve reference‐class ionization chambers, as defined in the IAEA TRS‐398 Rev.1 and the AAPM TG‐51 Addendum, were selected to evaluate both inter‐model and intra‐model variability.^1^, ^3^ These included two chambers each of the following models: PTW 30013 and 31021 (PTW‐Freiburg, Germany), Exradin A12 and A1SL (Standard Imaging, USA), and IBA CC13 and FC65G (IBA Dosimetry, Germany).

To account for fluctuations in linear accelerator output, an additional PTW 30013 chamber was used as an external monitor throughout the measurements. The specifications of each chamber are summarized in Table 1.

**TABLE 1:** Summary of the reference‐class ionization chambers used in this study.

Chamber type	Serial number	Usage history	Year of manufacture	Cavity volume (cm^3^)
Exradin A12	XA151635	Annually	2014	0.64
Exradin A12	XA210531	Annually	2021	0.64
Exradin A1SL	XW210541	Annually	2021	0.053
Exradin A1SL	XW240290	New Chamber	2024	0.053
IBA CC13	11491	Monthly	2012	0.13
IBA CC13	12305	Monthly	2013	0.13
IBA FC65‐G	4764	Monthly	2020	0.65
IBA FC65‐G	4769	Monthly	2020	0.65
PTW 30013	12238^a^	Weekly	2022	0.6
PTW 30013	12239	Monthly	2022	0.6
PTW 30013	3800	Annually	2009	0.6
PTW 31021	143708	Annually	2022	0.07
PTW 31021	143709	Annually	2022	0.07

^a^ Indicates the external monitor chamber.

Chambers with a sensitive volume greater than 0.6 cc were classified as “large‐volume,” while those with smaller volumes were categorized as “middle‐volume.” These models are commonly used for reference dosimetry of high‐energy photon beams in clinical practice, as reported in a recent survey on dosimetry practices conducted by Muir et al.^5^

### 2.2 Pre‐irradiation measurements

The electrometer (RTQM, RT521R2, Japan) was installed in the linac control room and powered on more than 14 h prior to the measurements under controlled environmental conditions (room temperature: 25  ±  1°C; relative humidity: approximately 50%). This ensured that the electrometer reached thermal equilibrium by the time of measurement. Similarly, the ionization chambers and the water phantom (MRWP400, MU Lab, Japan) were stored in the linac treatment room at least 14 h before measurement to achieve thermal equilibrium.

One hour before the measurements, the water temperature was controlled to 25.00  ±  0.01°C using a temperature control system (MWTP200, MU Lab, Japan), ensuring stable temperature conditions for both the chamber cavity and the surrounding water. The temperature control system continuously circulates water within the phantom to maintain a constant temperature, with a catalog‐specified accuracy of ±0.1°C. The validity of the system was confirmed by an independent digital thermometer, which consistently indicated a temperature of 25.0°C.

**FIGURE 1:**
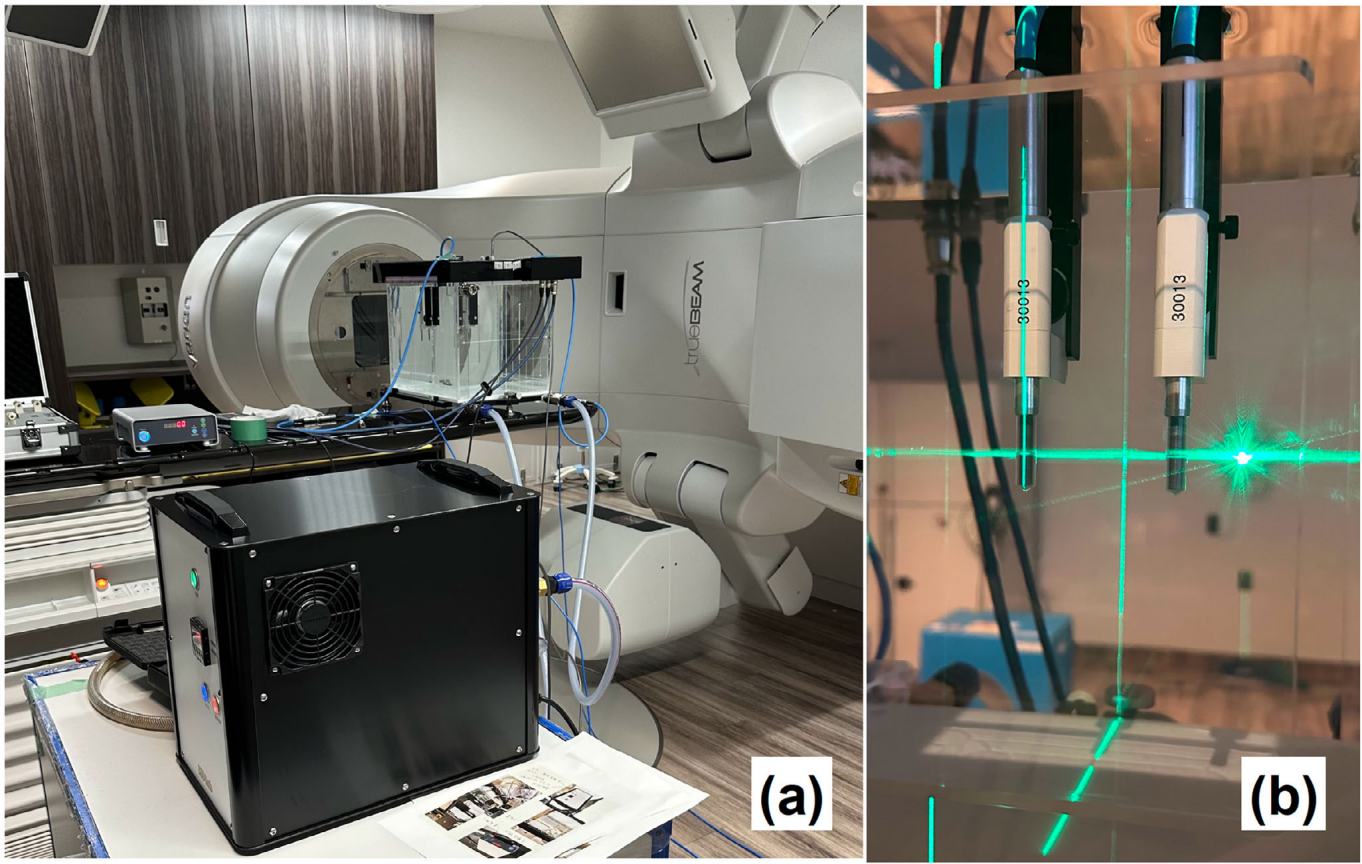
Experimental setup for pre‐irradiation measurements under thermal equilibrium conditions. (a) Measurement system comprising a water phantom (MRWP 400) and a temperature control unit (MWTP 200). (b) Two PTW 30013 ionization chambers: one positioned on the central beam axis, and the other placed laterally as an external monitor chamber beside the test chamber.

Pre‐irradiation measurements were performed using a 10 MV photon beam from a TrueBeam linac (Varian Medical Systems, USA). The linear accelerator was powered on 1 h prior to the start of measurements. In addition, 500 monitor units (MU) were delivered as a warm‐up before placing the phantom to ensure output constancy. The test ionization chambers were positioned in the water phantom at a depth of 10 cm along the central beam axis. The external monitor chamber (PTW 30013) was placed 3 cm lateral to the central beam axis, at the same depth as the test chamber (Figure 1). The external monitor chamber was fixed in position throughout the entire measurement session, and a constant bias voltage of −300 V was applied to the central electrode. Each test chamber was irradiated 30 times with 100 MU at 20‐s intervals using a bias voltage of −300 V. The same procedure was repeated after switching the polarity to +300 V. Voltage was applied to the central electrode: negative bias collected positive charge, and positive bias collected negative charge. Between chamber exchanges, each chamber was left in the water phantom for at least 10 min to allow temperature stabilization. Bias voltages for both polarities were applied immediately before measurement, and the leakage current was confirmed to be below 20 fA. Atmospheric pressure was monitored during all measurements. The maximum fluctuation during the 30 consecutive irradiations (conducted over approximately 15 min) was 0.2 hPa, which corresponds to a change of approximately 0.02% in the k_TP_ correction factor. As this variation was negligible, no k_TP_ correction was applied in this study.

The measured outputs from each test chamber were normalized to the output of the external monitor chamber, using the value at the 30th irradiation as the normalization point. Measurement stability was assessed in accordance with the AAPM WGTG51 Report No. 374. The point at which both of the following conditions were first satisfied was defined as the point of stabilization^6^:

1. The variation in chamber readings was within ±0.1%.
2. No systematic trend was observed in the readings.

The number of irradiations required to reach the stabilization point (denote as *N*) was defined as the optimal number of pre‐irradiation exposures for each chamber.

### 2.3 Determination of required pre‐irradiation dose

The required pre‐irradiation dose, *D* [cGy], for each chamber was calculated using the optimal number of exposures, 𝑁, as defined in Section 2.2, according to the following equation:

(1) $$\begin{array}{cc} D\;\left[cGy\right] & =100\left[MU/exposure\right]\times N\left[exposures\right]\\ & \times DMU\left[cGy/MU\right]\times TMR\left(10\;cm\right) \end{array}$$

Here, *N* represents the number of pre‐irradiation exposures required to stabilize the chamber response. *DMU* refers the dose per monitor unit at the reference point (*d*_max_), and was set to 1 cGy/MU based on institutional calibration. TMR(10 cm), the tissue‐maximum ratio at depth of 10 cm, was set to 0.85 based on in‐house measurements conducted at our institution.

## 3 RESULTS

**FIGURE 2:**
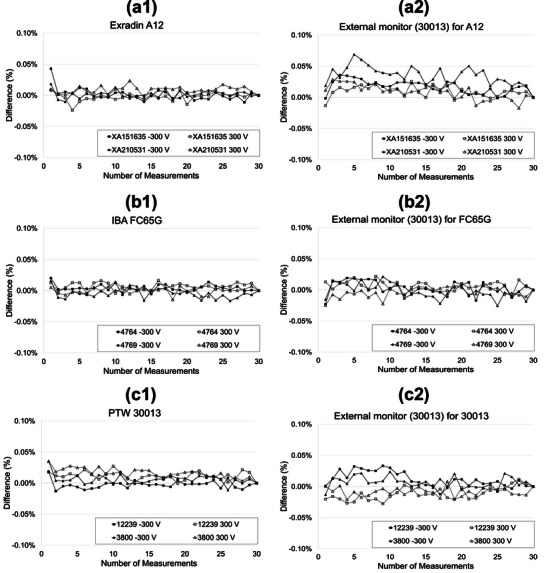
Normalized readings of large‐volume ionization chambers over 30 consecutive irradiations. (a) Exradin A12; (b) IBA FC65G; (c) PTW 30013 (two chambers each). Bias voltage: ± 300 V. Panels (a2)‐(c2) show the readings from the external monitor chamber (PTW 30013) under corresponding conditions, operated at a constant bias of −300 V.

**FIGURE 3:**
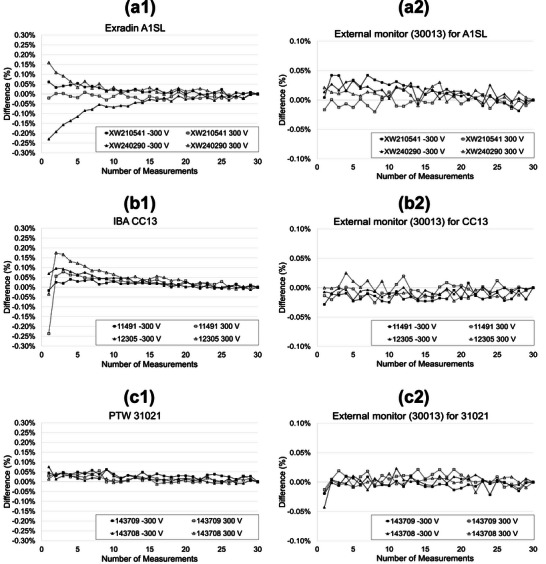
Normalized readings of middle‐volume ionization chambers over 30 consecutive irradiations. (a) Exradin A1SL; (b) IBA CC13; (c) PTW 31021 (two chambers each). Bias voltage: ± 300 V. Panels (a2)‐(c2) show the readings from the external monitor chamber (PTW 30013) under corresponding conditions, operated at a constant bias of −300 V.

Figure 2 presents the normalized output readings for the large‐volume ionization chambers Exradin A12, IBA FC65G, and PTW 30013 (normalized to the 30th irradiation). Panels a1, b1, and c1 show the readings from the test chambers A12, FC65G, and 30013, respectively, while panels a2, b2, and c2 display the corresponding readings from the external monitor chamber for each measurement. The PTW 30013 chamber was used as the external monitor throughout the experiment, operated at ‐300 V and held in a fixed position. In all three test chambers, the first reading was approximately 0.03% higher than the stabilized value, with deviations remaining within ± 0.05%. The A12 and 30013 chambers exhibited higher initial readings at ‐300 V compared to +300 V, whereas the FC65G showed similar values regardless of polarity. No clear polarity‐dependent trends were observed. Figure 3 shows the normalized output readings for the medium‐volume ionization chambers Exradin A1SL, IBA CC13, and PTW 31021 (normalized to the 30th irradiation). Panels a1, b1, and c1 show the readings from the test chambers A1SL, CC13, and 31021, respectively, while panels a2, b2, and c2 display the corresponding readings from the external monitor chamber for each measurement. As in Figure 2, the PTW 30013 was used as the external monitor chamber throughout. For the Exradin A1SL chamber (XW210514), readings for both +300 V and ‐300 V polarities remained within ± 0.1% from the first measurement; however, systematic trends persisted until stabilization around the 20th irradiation. The other A1SL unit (XW240290) reached stability within ±0.1% at the 3rd irradiation with +300 V and 6th with −300 V, with systematic variation converging around the 25th irradiation in both cases.

For the IBA CC13 chamber (11491), readings stabilized at the 2nd irradiation with +300 V and at the 1st irradiation with −300 V. Systematic trends diminished by the 20th irradiation. The second unit (12305) showed stability at the 7th irradiation with +300 V and the 1st with −300 V, with convergence around the 25th irradiation.

The PTW 31021 chamber exhibited deviations within ± 0.1% from the first measurement, and systematic behavior stabilized around the 10th irradiation.

Among the middle‐volume chambers, polarity effects varied by model. The Exradin A1SL showed notable inter‐unit variability. For both IBA CC13 units, the initial reading with +300 V was clearly lower than the stable value, while readings with −300 V were stable from the first irradiation. In contrast, the PTW 31021 chamber exhibited no polarity‐dependent differences.

Regarding usage history, only the Exradin A1SL (XW240290) was newly manufactured, and its response behavior differed from the older unit (XW210514). For the other chambers, no substantial differences were observed with respect to usage duration or frequency.

The values from panels a2 to c2 in Figures 2 and 3 represent the readings from the external monitor chamber, measured with a −300 V bias applied and the PTW 30013 held in a fixed position. These variations reflect both linac output fluctuations and measurement uncertainties. Although some values exceeded ± 0.05%, most remained within this range, indicating stable system performance.

Table 2 summarizes the number of irradiations required for output readings to fall within ± 0.1%, the corresponding cumulative doses, the optimal number of pre‐irradiation exposures (determined by convergence of systematic trends), and the resulting required doses. For the large‐volume chambers, although readings were within ± 0.1% from the first irradiation, a slight overshoot was observed. Therefore, the optimal number of pre‐irradiation exposures was set to 1, corresponding to a dose of 0.85 Gy, as calculated using Equation (1).

**TABLE 2:** Number of irradiations and corresponding doses (Gy) required for measurement stability in each reference‐class chamber.

Chamber type	Serial number	Times below 0.1%	Dose below 0.1% (Gy)	Stabilization time (irradiations)	Stabilization dose (Gy)
Exradin A12	XA151635	0	0	1	0.85
Exradin A12	XA210531	0	0	1	0.85
Exradin A1SL	XW210541	0	0	20	17
Exradin A1SL	XW240290	5	4.25	25	21.25
IBA CC13	11491	1	0.85	20	17
IBA CC13	12305	6	5.1	25	21.25
IBA FC65‐G	4764	0	0	1	0.85
IBA FC65‐G	4769	0	0	1	0.85
PTW 30013	12239	0	0	1	0.85
PTW 30013	3800	0	0	1	0.85
PTW 31021	143708	0	0	10	8.5
PTW 31021	143709	0	0	10	8.5

For the middle‐volume chambers, the optimal number of exposures ranged from 20 to 25 for the Exradin A1SL and IBA CC13, corresponding to doses of 17.0‐21.25 Gy. The PTW 31021 chamber required 10 exposures, equivalent to a dose of 8.5 Gy.

## 4 DISCUSSION

This study aimed to isolate and evaluate the intrinsic stability of ionization chambers by minimizing uncertainties commonly associated with reference dosimetry. This was achieved through strict temperature and humidity control, as well as compensation for linac output fluctuations using the external monitor chamber.

Previous reports have recommended pre‐irradiation doses of approximately 10 Gy, noting that measurement errors up to 1% may occur if this step is omitted, depending on the chamber model.^1^, ^3^ However, these studies typically assessed entire dosimetry systems without isolating individual chamber behavior, and were often conducted under conditions where system‐wide uncertainties – such as temperature fluctuations and linac variability – were not excluded.

In our study, ionization chambers were classified by sensitive volume into large‐ and middle‐volume groups and assessed individually. All large‐volume chambers (Exradin A12, IBA FC65G, and PTW 30013) exhibited deviations within ± 0.05% from the first irradiation, suggesting that pre‐irradiation may not be necessary under stable clinical conditions.

In contrast, the middle‐volume chambers (Exradin A1SL, IBA CC13, PTW 31021) showed greater variability, with deviations of up to ± 0.25%. Some chambers required more than 20 irradiations – approximately 20 Gy – to achieve stable output. Notably, our stability criterion was relatively stringent, requiring both deviation within ± 0.1% and the absence of systematic trends. According to the AAPM WGTG51 Report 374, fluctuations within ± 0.1% are considered acceptable when multiple readings are averaged. ⁶ Based on this, a pre‐irradiation dose of approximately 5 Gy may be sufficient in many cases (Table 2).

Polarity effects were also examined. While previous literature suggests that pre‐irradiation is required following a polarity change,^6^ significant polarity dependence was observed in only a subset of middle‐volume chambers, indicating that polarity sensitivity may vary across chamber models and individual units. For example, one IBA CC13 unit exhibited an initially low reading with +300 V, whereas the −300 V condition was stable from the first irradiation. These results suggest that polarity sensitivity varies by model and even between individual chambers.

Whereas previous study^4^ used Co‐60, our experiment employed a linear accelerator, necessitating continuous output monitoring with an external monitor chamber. As shown in Figures 2 and 3, the output readings of the external monitor chamber normalized to the 30th irradiation demonstrated fluctuations within ± 0.1% across all time points, confirming the stability of the linac itself. Therefore, the observed output variations can be attributed primarily to chamber behavior, rather than beam‐level fluctuations. This further supports our conclusion that the actual magnitude of pre‐irradiation‐induced variability is substantially lower than the previously reported 1% under controlled conditions. However, since the required pre‐irradiation dose for the ionization chambers was not zero, it is presumed – consistent with the IAEA TRS‐398 – that mechanisms such as charge imbalances among internal materials and variations in leakage current before and after irradiation continue to contribute, albeit to a limited extent.

A key strength of this study is the experimental determination of the pre‐irradiation dose required for ionization chambers, minimizing external uncertainties while considering clinically relevant factors such as chamber type, polarity, usage history, and aging. By clarifying the dose needed for measurement stability, the results support the rationalization of reference dosimetry procedures. However, some limitations should be acknowledged. First, although variations among chambers of the same model were observed – possibly due to usage or aging – the sample size was small, warranting larger‐scale studies. Second, only reference‐class chambers were investigated, so the applicability to small‐volume detectors such as microchambers remains unclear. Third, electrometer and cable effects were excluded to isolate chamber behavior; however, these components may contribute to uncertainty in clinical settings. Potential contributing factors include variations in storage‐related temperature and humidity, thermal equilibration between the room and water phantom, sudden atmospheric pressure changes, and air conditioning conditions in the treatment room. Fourth, only a single 10 MV photon beam from a TrueBeam linac was tested, and further validation across different beam energies, linac models, and clinical environments is necessary.

## 5 CONCLUSION

In reference dosimetry, ensuring sufficient output stability of the ionization chamber is essential. Based on our measurements, the required pre‐irradiation dose was approximately 0.85 Gy for large‐volume chambers and up to 21.25 Gy for middle‐volume chambers. This study focused exclusively on the behavior of the ionization chambers themselves under strictly controlled conditions that excluded system‐level uncertainties. Consequently, the previously reported output variation of up to 1% was not observed; instead, output fluctuations for all large‐volume chambers were within ±0.05%. These findings indicate that for certain large‐volume chambers, pre‐irradiation requirements may be substantially lower than previously assumed when measurements are conducted under highly stable and controlled conditions. While our results do not contradict existing recommendations such as those in IAEA TRS‐398 or AAPM TG‐51, they suggest that further evidence‐based refinement of pre‐irradiation practices may be possible in the future. These observations may contribute to streamlining and standardizing reference dosimetry procedures, provided that chamber‐ and condition‐specific validation is performed.

## AUTHOR CONTRIBUTIONS

T. Yamada, K. Mayumi, T. Nishikata, and N. Kinoshita designed the study and performed the experiments. S. Tanabe provided guidance on the study's content. T. Sakai, H. Sakai, T. Oka, H. Ishikawa, and T. Kanazawa supervised the study and reviewed the manuscript.

## CONFLICT OF INTEREST STATEMENT

The authors have no conflict of interest to declare.

## DISCLOSURE

This manuscript was prepared with the assistance of a generative AI language model (ChatGPT, OpenAI). The tool was used to refine language, improve clarity, and support translation during manuscript preparation. All AI‐generated content was reviewed and approved by the authors, who take full responsibility for the final version of the manuscript.
